# Evaluating Geospatial Sampling Frames with a Novel Field Census for a Malaria Household Survey in Artibonite, Haiti

**DOI:** 10.4269/ajtmh.23-0891

**Published:** 2024-08-13

**Authors:** Karen E. S. Hamre, Amber M. Dismer, Nishant Kishore, Anyess Travers, Kathleen McGee, Bernadette Fouché, Luccène Désir, Kathleen Holmes, Gregory S. Noland, Jean Frantz Lemoine, Michelle A. Chang

**Affiliations:** ^1^ Malaria Branch, Division of Parasitic Diseases and Malaria, Center for Global Health, Centers for Disease Control and Prevention, Atlanta, Georgia;; ^2^ CDC Foundation, Atlanta, Georgia;; ^3^ Emergency Response and Recovery Branch, Division of Global Health Protection, Center for Global Health, Centers for Disease Control and Prevention, Atlanta, Georgia;; ^4^ Population Services International/Organisation Haïtienne de Marketing Social pour la Santé, Peguy-ville, Haiti;; ^5^ The Carter Center, Atlanta, Georgia;; ^6^ Programme National de Contrôle de la Malaria, Ministère de la Santé Publique et de la Population, Port-au-Prince, Haiti

## Abstract

The Ministry of Public Health and Population in Haiti is committed to malaria elimination. In 2017, we used novel methods to conduct a census, monitor progress, and return to sampled households (HH) before a cross-sectional survey in La Chapelle and Verrettes communes in Artibonite department (“the 2017 Artibonite HH census”). Geospatial PDFs with digitized structures and basemaps were loaded onto tablets. Enumerators captured GPS coordinates and details of each HH and points of interest. The census used 1 km^2^ enumeration areas (EAs) to draw a representative sample. Three remote sampling frames were compared with the 2017 Artibonite HH census. First, 2003 census EAs with 2012 population estimates from the Haitian Institute of Statistics and Informatics were standardized to the study EAs. The second sampling frame used the 2016 LandScan^TM^ population estimates and study EAs. The third sampling frame used structures ≥3 m^2^ manually digitized using Maxar satellite images. In each study EA, 70% of structures were estimated to be inhabited with 4.5 persons/HH. The census identified 33,060 inhabited HHs with an estimated population of 121,593 and 6,126 points of interest. Using daily coverage maps and including digitized structures were novel methods that improved the census quality. Manual digitization was closest to the census sampling frame results with 30,514 digitized structures in the study area. The LandScan^TM^ method performed better in urban areas; however, it produced the highest number of HHs to sample. If a census is not possible, when feasible, remotely digitizing structures and estimating occupancy may provide a close estimate.

## INTRODUCTION

For studies in many settings, high-quality granular household (HH)-level data are unavailable, and practitioners select from a variety of other sampling methods because of the significant financial resources needed to conduct a census, available time, geographic area, and logistical complexity. Using census data as a sampling frame for a representative survey is considered the gold standard and is used for Demographic and Health Surveys when a recent census has been conducted.^1^^–^^4^ We outline a novel approach for creating an accurate sampling frame before a cross-sectional malaria HH survey in Artibonite department, Haiti, in 2017. Prior to the Artibonite HH malaria survey, the most recent national census in Haiti was conducted in 2003.^5^ Although spatially referenced enumeration areas (EAs) from the 2003 census with population projections in 2012 were available, the 14-year-old census was outdated for the planned sampling approach.^6^ This article describes the 2017 Artibonite HH census undertaken for this survey, the creation of the spatial sampling frame, and unique geospatial monitoring methods used in the survey. We also compare this novel approach with three sampling frames created using remote population estimates to evaluate how these sampling frames would have impacted the Artibonite HH survey sample size, and hence, results.

Haiti and the Dominican Republic, which share the island of Hispaniola, are the only remaining malaria-endemic countries in the Caribbean. *Plasmodium falciparum* is the dominant parasite species, responsible for more than 99% of cases detected on the island in 2017 (the year this census occurred).^7^^,^^8^ In Hispaniola, the parasite is still susceptible to chloroquine, a relatively inexpensive and safe antimalarial drug, which remains the first-line treatment of uncomplicated malaria in this region.^9^^,^^10^ The main vector of transmission, *Anopheles albimanus*, is primarily exophilic with zoophilic tendencies and is relatively inefficient at transmitting malaria.^7^ The technical feasibility of malaria elimination on the island was previously evaluated and supported by both governments, as evidenced by all other Caribbean islands having successfully eliminated malaria within their respective borders.^11^

**Figure 1.**
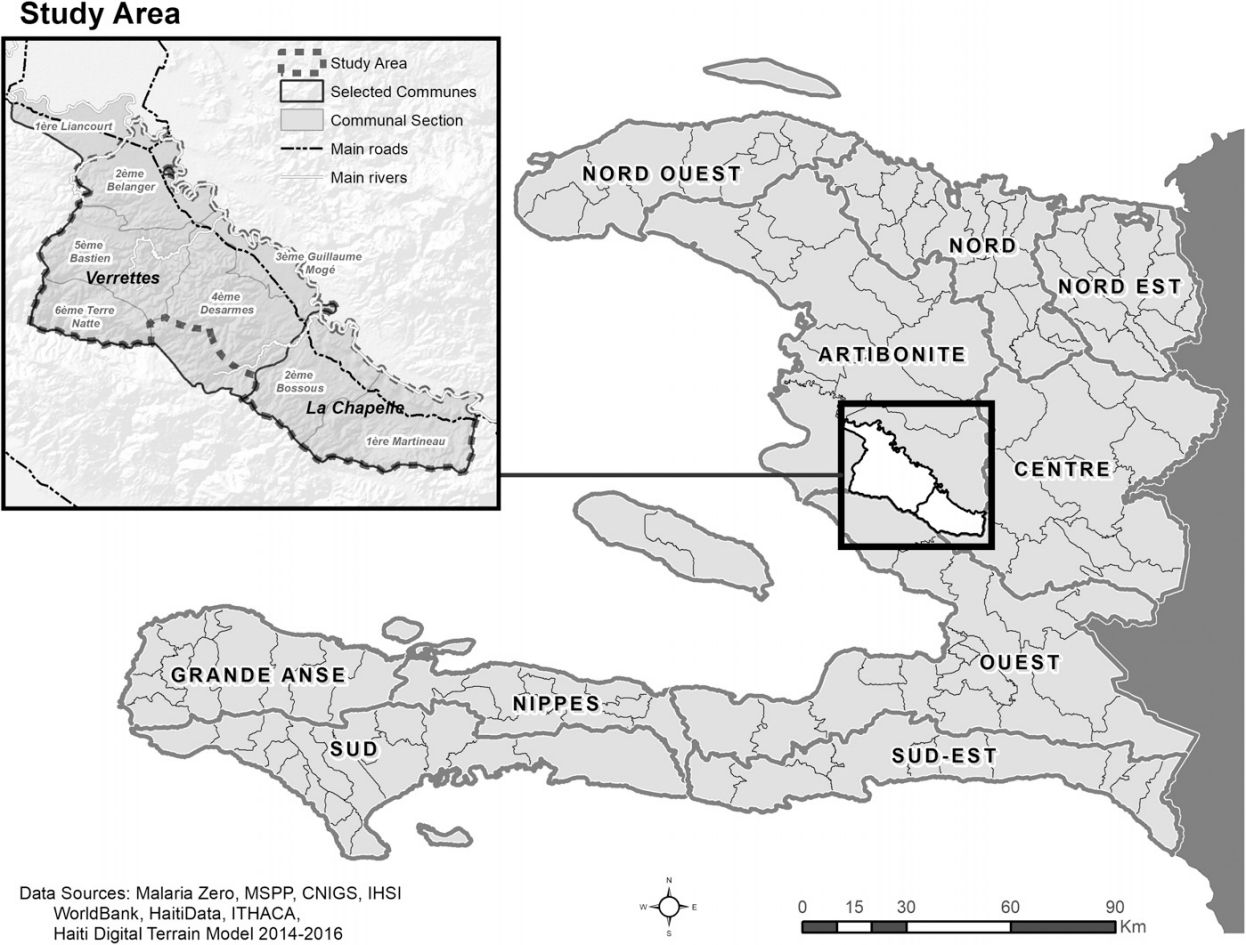
Study area. Data sources: Malaria Zero, Ministry of Public Health and Population (MSPP), National Center for Geospatial Information (CNIGS), Haitian Institute of Statistics and Information (IHSI), World Bank, Haiti Data, Information Technology for Humanitarian Assistance, Cooperation and Action (ITHACA). Haiti Digital Terrain Model 2014–2016.

Surveillance data indicate that 98% of confirmed malaria cases on Hispaniola occur in Haiti, where 18,843 confirmed cases were reported in 2017.^8^ Identifying and focusing on locales and populations most at risk is paramount to achieving elimination. The Malaria Zero consortium partners, including the Ministry of Population and Public Health (Ministère de la Santé Publique et de la Population [MSPP]) of Haiti, united to accelerate malaria elimination efforts on the island.^10^^,^^11^ To characterize foci of malaria transmission to inform the elimination strategy, a cross-sectional HH survey was developed and conducted in La Chapelle and Verrettes *communes* (districts) in the Artibonite department from July through October 2017, as described by Hamre et al.^12^ The study area is landlocked with valleys and mountainous terrain; the Artibonite River flows through it (Figure 1). This study area was selected for both its accessibility after Hurricane Matthew made landfall in October 2016 that prevented access to the original study locations in the Tiburon Peninsula, and for its relatively high number of malaria cases (2016 incidence of malaria in these communes were 3.9 and 2.1 cases per 1,000 persons, respectively) compared with the overall incidence across the nation (2.0 per 1,000 persons in 2016; MSPP, unpublished data). To evaluate transmission in rural, hard-to-reach populations living outside the main city centers of La Chapelle and Verrettes, we determined that a highly accurate denominator and spatially representative sample was required to document the potentially small malaria foci to target to accelerate the island toward malaria elimination.

Many geographic information system machine-learning models exist to identify buildings from high resolution satellite imagery using either fully automated or semiautomated approaches (e.g., humans identify a sample that is used for automation). Additionally, different methods exist for estimating the number of multifamily structures and population.^13^ It is a rare opportunity to evaluate the differences between a census covering the entire study area and other GIS methods. To accomplish this, three remote spatial sampling frames were compared with the 2017 Artibonite HH census sampling frame. These models included: 1) 2003 census areas with 2012 population projections interpolated to a grid, 2) 2016 LandScan^TM^ world population grid, and 3) digitized structures with an estimate of 70% structure being HHs with people living in them. For the third model, each HH was estimated to have 4.5 people as designated by the last Enquête Mortalité, Morbidité et Utilisation des Services (EMMUS V; French for Survey of Mortality, Morbidity, and Utilization of Services).^14^^,^^15^ For comparison, grids were classified into three strata using each sampling frame method and the spatial sample was drawn.

The census and HH survey protocol (#6821) was reviewed and approved by the Haitian Bioethics Committee and by the Centers for Disease Control and Prevention’s Institutional Review Board.

## MATERIALS AND METHODS

Before conducting the census, high-resolution Maxar satellite imagery (©2022 Maxar Nextview) acquired in 2016 from February to December in La Chapelle and Verrettes were imported into ArcGIS Desktop version 10.5 (ESRI, Redlands, CA).^16^ LandScan™ world population 2016 grid cell boundaries of 1 km^2^ were overlaid on the imagery and numbered to serve as census enumeration areas for the 2017 Artibonite census (grid cell EAs).^15^ Five students used the 1-km^2^ grid cells to digitize manually all structures with rooftops ≥3 m^2^ in the study area; these structures were reviewed by the GIS analyst and served as a guide for the enumerators. In total, the five students spent 138.5 hours to digitize structures in Verrettes (surface area: 356.7 km^2^) and La Chapelle (surface area: 143.6 km^2^), inclusive of 2 hours per person for training.^5^ Structures and known points of interest were used to create maps and to develop the deployment plans for the enumeration teams.

Meetings with local government officials, police, and community leaders were held to discuss the logistics and upcoming Malaria Zero activities in the area and answer any questions. With the aid of community leaders, local guides were identified to help navigate remote terrain and to ensure that all areas with structures were reached. Often, these guides worked as community health workers and were identified jointly with the National Malaria Control Program, the Artibonite department’s Ministry of Health staff, Population Services International, and The Carter Center. Thirty-five teams of two were deployed to complete the census, which was estimated to take 16 days. The maps with the digitized structures were loaded onto handheld tablets (BLU Studio 7.0 II) as geospatial PDFs with both satellite and OpenStreetMap base maps to aid in navigation and viewed in PDF Maps^TM^ application. Census data collection occurred in CommCare on Global System for Mobile Communications (GSM)-enabled tablets for near real-time data synchronization. Enumerators were trained to visit each grid cell EA and systematically assess the area using the digitized structures as a guide, recognizing where homes may have been built, or taken down, since the time of the satellite imagery. For each residential structure, global positioning system (GPS) coordinates were captured. If someone over age 15 years was available to answer questions about the residence, additional information was collected including the number of families who lived in the structure, the head of HH name for each family, the HH size, and a brief description of any unique or identifying features of the residential structure. No revisits were conducted to structures; either neighbors over 15 years old answered questions about the structure’s inhabitants or the location was recorded as an HH (inhabited/uninhabited) and an estimated 3.67 (from study population) was imputed for inhabited structures. The name, description, and GPS data from points of interest (POIs) were also collected to aid in operations and relocating sampled HHs in future surveys described by Hamre et al.^17^ POIs included health facilities, pharmacies, churches, schools, markets, bridges over rivers, police stations, and transportation hubs. Enumerators worked with local guides to visit EAs without digitized structures to identify newly built HHs and POIs since the date of the satellite images or to verify if none existed. For digitized structures visited that were not HHs or POIs, enumerators captured the GPS coordinates and a descriptor (e.g., abandoned house, house under construction, empty field) such that the coverage maps would indicate the location was indeed visited. The teams could only record a GPS point directly on the tablet if the accuracy was ≤5 m. When the tablet accuracy was >5 m, teams used a handheld GPS device (Garmin GPSMAP 62sc Handheld Navigator or Garmin eTrex; Garmin, Olathe, KS) to record and manually enter coordinates into CommCare. Teams also completed a standardized end-of-day report on the tablet to document any issues they experienced throughout the day.

Nightly, field data were uploaded to the CommCare server and reports of operational challenges (e.g., Internet connectivity for data transfer, access issues such as flooded roads, security issues) were reviewed and addressed by the field managers during in-person meetings. Additionally, the updated data were exported from CommCare, imported into Quantum GIS version 2.8, and overlaid on the digitized structures to identify completed EAs and missed areas. Updated coverage maps were created and shared with enumerators and supervisors every morning either on paper or via encrypted WhatsApp messages for feedback and to inform the return to a specific grid cell EA, if necessary. A Microsoft Excel dashboard that queried data from CommCare was created to monitor progress; the dashboard contained the cumulative numbers of HHs, estimated number of persons, and numbers of POIs enumerated.

**Figure 2.**
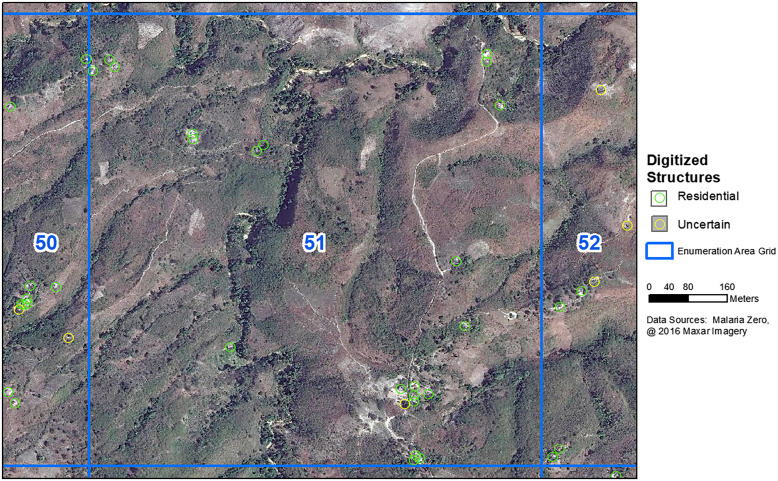
Digitized structures. Data sources: Malaria Zero, 2016 Maxar Imagery.

On the basis of operational feasibility for the planned HH survey, the final study area was trimmed before the commencement of census activities (Figure 1). High-resolution satellite imagery from Maxar was used for digitization, field census maps, and overlaying data for validation efforts (Figure 2).^16^

The data collected during the census were used to create new maps for the HH survey, with the sampled HHs and recently identified POIs to serve as guides for deployment. In addition, the details of the HH, including the GPS coordinates, head of HH name, and brief description of the HH and structure were loaded onto the tablets in CommCare for all sampled HHs to aid in returning to the sampled HH, recognizing multiple HH may reside in the same structure.^16^^,^^17^

The number and location of digitized structures was compared with structures identified in the 2017 Artibonite HH census. In ArcGIS, the nearest geodesic distance from each digitized structure to each censused structure was calculated. A cutoff distance of >30.4 m was used to identify structures that were spatially too far apart to be the same structure.

A methods comparison was conducted between calculations of population and HH estimates within grid cells that were used for spatial sampling in the HH survey (see Supplementary Table 1). With the increasing availability of population datasets available from LandScan^TM^ (1-km resolution), WorldPop, and bottom-up estimates from GRID3, efforts such as this comparison provide real-world information on reliability back to the global community.^13^^,^^15^^,^^18^ Therefore, the 2017 Artibonite census was used as the gold standard and compared with other estimates.

To create the first remote spatial sampling frame, the 2003 enumeration areas (2003 EA) provided by the Haitian Institute of Statistics and Informatics (IHSI) were resampled to the 2017 Artibonite 1-km^2^ grid cell surface using areal interpolation.^5^^,^^19^ To do this, the 2003 EAs and the 2017 Artibonite grid cell surface were each projected to the Universal Trans Mercator Zone 18N coordinate reference system based on the 1984 World Geodetic Survey. For each grid cell, the proportional population, HHs, and structures were calculated based on the area of the 2003 EA inside each grid cell. For example, if 3% of the 2003 EAs area fell within grid cell 1, then 3% of the population from 2012 was allocated. This method relies on the assumption that population is evenly distributed within a 2003 EA and within a grid cell. These communes were highly forested and did not have large lakes; therefore, no geospatial masks were applied to exclude a portion of the commune from being populated. To create the second remote spatial sampling frame, the 2016 LandScan^TM^ population dataset’s 1-km^2^ grid cell—the same as the 2017 Artibonite grid cell surface—was used. Because LandScan^TM^ population datasets do not include the estimated number of structures per grid cell, an estimate was calculated with 4.5 persons per HH based on the 2012 EMMUS V.^14^ To create the third remote spatial sampling frame, the number of digitized structures was aggregated to the 2017 Artibonite 1-km^2^ grid cell surface. On the basis of past field work in Artibonite in 2014 (water and sanitation survey conducted, data unpublished), it was estimated that approximately 70% of structures manually digitized by the team and ≥3 m^2^ were HHs. To estimate the population of the 20,801 HHs, the 2012 EMMUS V estimate of 4.5 persons per HH was multiplied by the number of HHs.

For a comparison of spatial sampling methods, each grid cell in the four sampling frames was categorized as being in a large operational unit (OU) (>200 households; (Strata 1), medium OU (20–200 households; Strata 20, or small OU (<20 households; Strata 3). Ten percent of HHs in each grid cell in Strata 1, 20 HHs in each grid cell in Strata 2, and all HHs in Strata 3, plus a 5% oversample in Stratas 1 and 2, were calculated for each sampling method (Table 2, predicted sample).^17^ Sampling strata for each method was conducted in R Statistical Software v4.2.1 using the base R round function. Every grid cell was sampled; therefore, the final field sample was calculated using each method’s grid composition and the 2017 Artibonite census (Table 2, field sample). This spatial sampling method was used to ensure urban and rural populations were considered and to be able to detect malaria foci.

## RESULTS

### 2017 Artibonite field census.

A total of 40,444 structures in the communes of La Chapelle and Verrettes were digitized in advance of the census to inform activities. As mentioned, the study area was trimmed excluding the 1ère Liancourt and the southern part of 4ème Desarmes communal sections resulting in 30,514 total digitized structures (Figure 1; Table 1). After review, 314 duplicate points and 484 small structures (<3 m^2^) were excluded from the study area maps and analysis. All remaining structures (*N* = 29,716) were included for census verification efforts and the digitized method analysis.

**Table 1:** Comparison of digitized structures to Artibonite 2017 household census

Structure and HH Summaries	Study Area	Trimmed Area	Total
Total no. digitized	30,514	9,530	40,444
Duplicates	314	30	344 points
Small (<3 m^2^)	484	69	553
Structures	27,894	6,883	34,777
Uncertain structures (e.g., under construction, warehouses, rocks)	1,822	2,674	4,486
Total no. field census identified			
Total POIs	6,126		
Health structures (health facilities, *n* = 7; traditional doctors, *n* = 622; pharmacies, *n* = 17; immunization stations, *n* = 6)	652		
HH structures (kitchen, *n* = 1,680; toilet,* n* = 201; barn, *n* = 52; silo, *n* = 167)	2,100		
Village structures (bank, *n* = 40; cemetery, *n* = 59; church, *n* = 377; gambling house, *n* = 85; market center, *n* = 19; nightclub, *n* = 14; police station, *n* = 1; recreational facility, *n* = 99; restaurant, *n* = 34; school, *n* = 238; shop, *n* = 284; transport hub, *n* = 8)	1,258		
Bridge crossing a river	45		
Other (unspecified)	2,071		
HH Total	33,060		
Structure comparison (>30.4 m)			
No. in field and digitized*	33,002		
No. in field only (POI, *n* = 666; HHs, *n* = 3,147)	3,813		
No. digitized only (2,061 structures; 310 uncertain)	2,371		

HH = household; POIs = points of interest.

* Although only 29,716 structures were digitized, censused structures include multifamily HHs living in the same structure and multiuse buildings.

In the study area, the census was completed from June 1 through 22, 2017, during which a total of 33,060 households (with an estimated population of 121,593) were enumerated and included in the sampling frame for the household survey. Of the 33,060 HHs, 95.9% were available to provide population information totaling 116,631 people; for the remaining 1,352 HHs, each was estimated to have 3.67 persons (the calculated average HH size from HH with reported population information). The grid cell EA boundaries from the census served as OUs for the HH survey. There were 476 OUs within the final study area that had inhabited, residential households identified (162 populated in low strata, 283 in medium strata, and 31 in high strata). Of the 476 OUs, 51.4% had at least one imputed HH with an average of 2.99% of the population in those grid cells being attributed to imputation.

Additionally, 6,126 POI were collected during the census to help facilitate returning to the sampled HHs including health structures (e.g., formal health facilities, pharmacies, and traditional healers) and village features (e.g., market center, churches, schools) (Table 1). The final census dataset (33,060 HHs and 6,126 POIs) was compared with the digitized structures (29,716) to evaluate the remote work. A total of 3,813 structures were identified in the field that were >30.5 m away from digitized structures; 341 of these structures were geographically outside the study area. Similarly, 2,371 structures were identified via digitization with satellite imagery, however when the grid cell EAs where these structures were putatively located were visited, no structures were found.

### Methods comparison.

With the 2017 Artibonite field census data, we know the true number of HHs. If the 2016 LandScan^TM^ population dataset (the most current at that time) had been used, this sampling frame would have resulted in the highest number of HHs in the final dataset—12,701 HHs. In advance, teams would have expected 9,723 HHs but would have found an additional 2,978 HHs in Strata 3 in the field (Table 2).

**Table 2:** Estimates of numbers of structures, households, and population with final sample number, by sampling frame method

Sampling Frame Method	No. of Structures	Number of HHs	Population	Grid Composition			Predicted Sample*	Field Sample^†^
				3. Low Strata (<20 HHs)	2. Medium Strata (20–200 HHs)	1. High Strata (>200 HHs)		
2003 census enumeration areas with projected 2012 population	31,086	28,794	120,450	229 cells (26 cells have 0 HHs), 2,262 HHs	397 cells, 19,330 HHs	16 cells, 7,202 HHs	11,353	10,583
2016 LandScan^TM^ population with estimated 4.5 people per HH	51,547 (estimated)	36,083 (estimated)	162,375	387 cells (50 cells with 0 HHs), 3,080 HHs	205 cells, 10,502 HHs	50 cells, 22,501 HHs	9,723	12,701
Manually digitized structures with estimated 4.5 people per HH and 70% occupancy	29,716	20,801	93,605	383 cells (146 cells with 0 HHs), 1,936 HHs	245 cells, 13,752 HHs	14 cells, 5,113 HHs	7,612	9,008
2017 Artibonite census	35,371	33,060	121,593	328 cells (166 with 0 HHs), 1,433 HH	283 cells, 17,776 HH	31 cells, 13,851 HH	**8,818**	**8,818**

HH = household.

* No. of HHs expected in field.

^†^ No. HHs that would have been included following the methodology.

**Figure 3.**
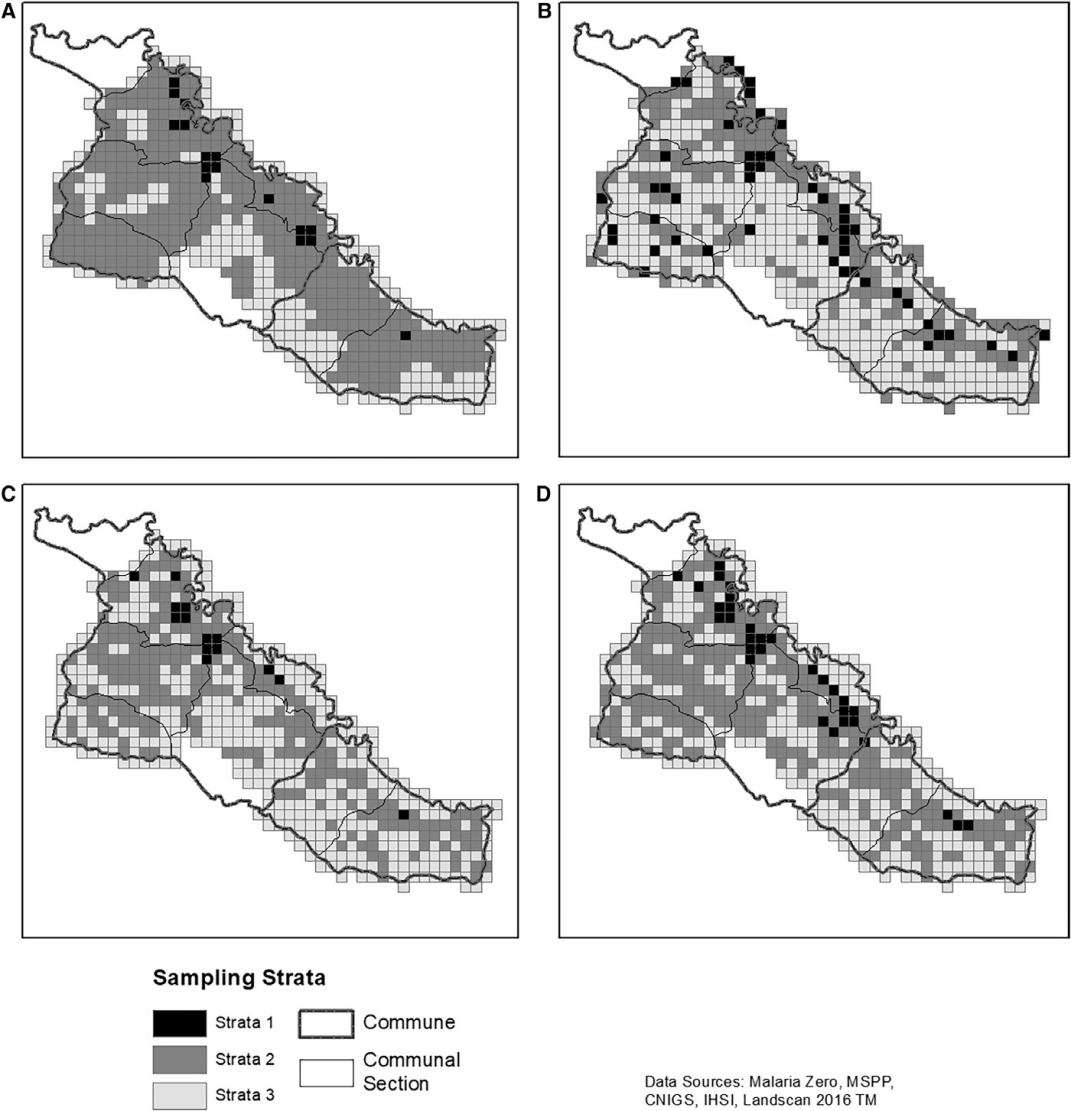
Sampling strata. (**A**) 2003 census enumeration areas with projected 2012 population. (**B**) 2016 LandScan^TM^ population estimates. (**C**) Estimated population derived from manual digitization of structures using Maxar imagery and national DHS household size of 4.5. (**D**) 2017 census population. Data sources: Malaria Zero, Ministry of Public Health and Population (MSPP), National Center for Geospatial Information (CNIGS), Haitian Institute of Statistics and Information (IHSI), and LandScan^TM^ 2016.

**Figure 4.**
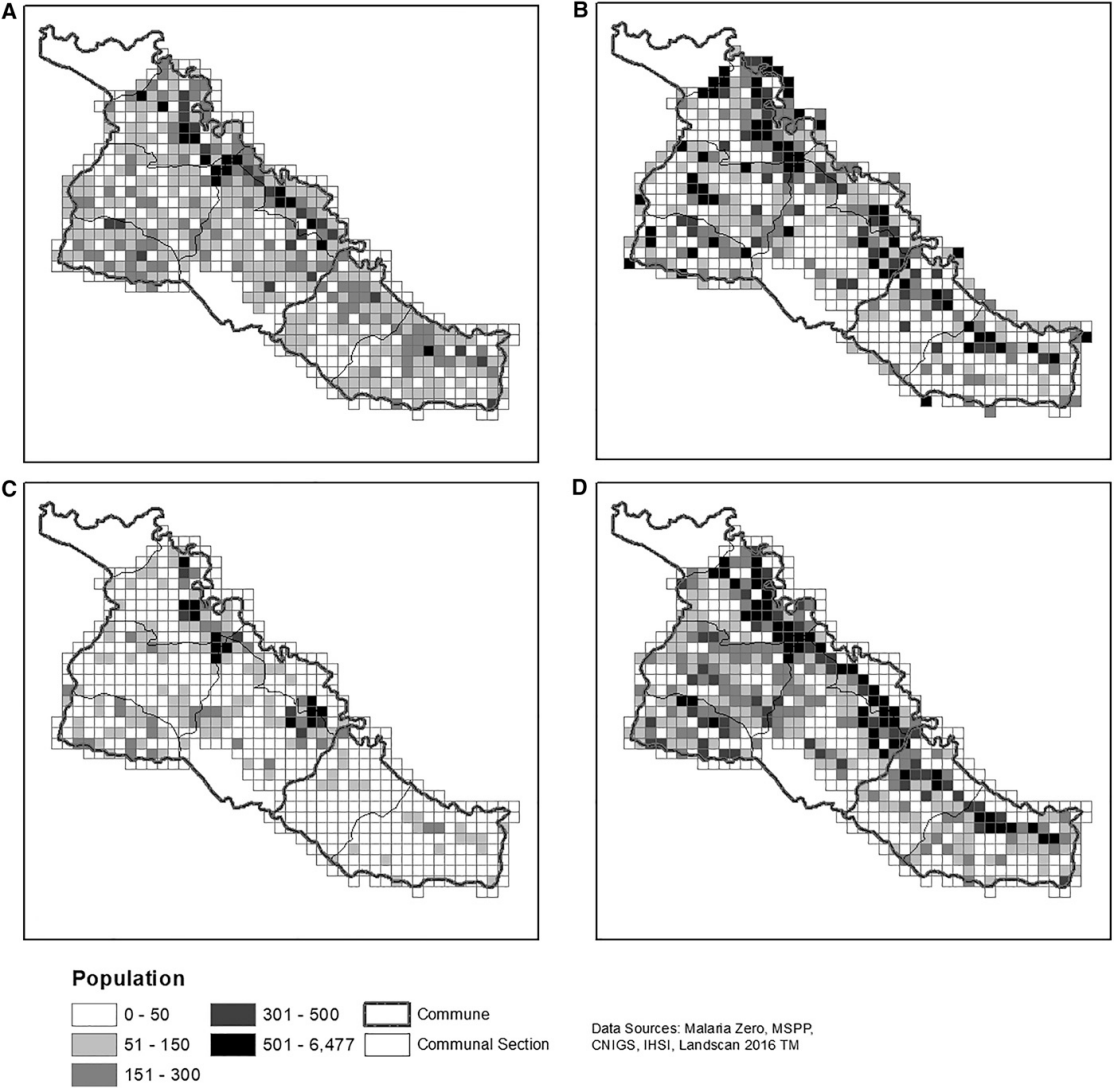
Population. (**A**) Absolute difference in population between the 2017 census and 2003 census enumeration areas with projected 2012 population; (**B**) Absolute difference in population between the 2017 census population and the 2016 LandScan^TM^ estimates; (**C**) Absolute difference in population between the 2017 census population and the digitized estimates; (**D**) 2017 census population. Malaria Zero, Ministry of Public Health and Population (MSPP), National Center for Geospatial Information (CNIGS), Haitian Institute of Statistics and Information (IHSI), and LandScan^TM^ 2016.

The sampling frame most similar to the 2017 Artibonite census grid’s distribution of HHs was generated by manually digitizing structures (Table 2; Figure 3C). When the manual digitization efforts were adjusted using the field census for Strata 3, an additional 1,396 HHs would have been visited with a total of 9,008 HHs. The digitization method most closely mirrored rural patterns of HHs and minimized population differences at the grid cell level (Figures 3C and 4C).

The sampling frame derived from the 2003 census enumeration areas projected with 2012 population estimates had the highest number of grid cells in Strata 2 and the least in Strata 1 (Table 2; Figure 3A). If this method had been used, 10,583 total HHs—770 less HHs than expected—would have been identified in the field.

Next, the absolute difference of the population per grid cell was calculated (Figure 4). In each of the three estimation methods, urban areas had higher population differences than rural areas when compared with the 2017 Artibonite census. The 2003 census EA method performed worst when examining the absolute difference in population for each cell (Figure 4A). Although the 2016 LandScan^TM^ method appeared to perform worse on the study area border (Figure 4B), this was because field teams did not enumerate structures outside the study area boundary, which split some EAs. The digitization method minimized the absolute differences of population within each cell (Figure 4C), especially in rural areas.

## DISCUSSION

A complete georeferenced census was successfully conducted in La Chapelle and Verrettes communes in Haiti to create the sampling frame for a population-based household survey. Digitizing structures in advance of the census activities improved the quality of the enumeration in the field. Digitization requires planning to secure high resolution satellite imagery, to train reviewers, and to identify structures. Manual digitization is feasible for small studies; however, it would not have been feasible for a national survey. Because of manual digitization efforts, the study area was able to be trimmed in advance (1re section Liancourt and 4eme Desarmes). Digitization has been used elsewhere, but researchers have not always conducted census validation activities. For example, for a survey to evaluate the effects of health system interventions in Mozambique, in the absence of recent census data, researchers digitized structures using satellite imagery and overlaid grids, which were then used as the primary sampling unit; in each sampled grid cell, the “random walk” methodology was used to select HHs to participate.^20^ Also in 2017, researchers in Guatemala were faced with a similar predicament while planning a HH survey to estimate prevalence of chronic kidney disease of unknown origin where the most recent census data available was from 2002.^21^ These researchers used Epicentre’s new Geo-Sampler tool to draw a simple random sample from structures digitized within a 15-m radius of the point. Although this eliminated the high cost of field enumeration, the tool is limited in forested areas due to tree coverage and in urban settings with multiple HHs residing in the same structure. Another study by Checchi et al. found that two researchers applying the same digitization methodology to residential and nonresidential structures of internally displaced persons camps in 10 countries presented a difference between <10% and 36% of enumerated structures.^22^

The authors recognize that a complete census may not always be feasible resource-wise. In these instances, alternative enumeration methods to generate the sampling frame should be considered. Although bottom-up population datasets are improving as GRID3 micro-censuses inform WorldPop estimates, there are still improvements to be made.^13^^,^^18^^,^^23^ Consideration to include a health economist to evaluate costs of the varying methods should be made during the planning stages of a study.

A manual structure digitization method with an adjusted structure to household ratio was the closest alternative GIS method to the census examined when creating gridded cells mirroring the true distribution of HHs and of the underlying population. Likely, digitization is also a cost-efficient solution, although cost of students to digitize was not explicitly evaluated. However, field logistics and associated costs would have been underestimated if using the digitization method’s predicted sample (*N* = 7,612 HHs). In terms of replicability to other settings, the structure to HH ratio should be modified based on available information. Depending on the size of the sampling frame, this ratio could be estimated relatively quickly and is expected to differ between urban and rural areas and sometimes across administrative units (e.g., across departments or regions).

The EAs from 2003 did account for geographic terrain changes and had an urban/rural classification label. Typically, census EAs are created to have approximately either the same number of HHs or approximately the same total population; the 2003 EAs did not have the same population size, with an average across the study area of 659 people per EA (interquartile range: 297 people). Therefore, some error most likely was introduced into the comparison analysis as population was equally distributed when the 2003 population was transformed to fit the geographic boundaries of the 2017 study EAs.

Every HH had a possibility of being selected into the sample when using the Artibonite 2017 census method. For the three remote methods, each would still require a field sampling implementation plan to select the HHs to sample HHs for Stratas 1 and 2. The 2003 census EAs with projected 2012 population and 2016 LandScan^TM^ population only identify grids to sample in advance. The manual digitization method preidentifies cells and structures but may either misclassify structures or miss them in forested areas. All the remote methods may introduce error into the sample and result in some HHs having a zero probability of being included in the sample.

Providing visual spatial coverage feedback to the teams daily improved the quality of the census. These coverage maps illustrated areas where structures (e.g., potential HHs) were digitized in advance that were not yet visited (per reported GPS coordinates) in some EAs that teams had self-reported as complete. Although it is unclear why the teams thought they had visited all structures, having this GPS-confirmed coverage information updated daily influenced deployment strategies and improved the confidence in the census results.

Systematically collecting ancillary data, such as POIs and HH descriptors during the enumeration of HHs improved the validity of the subsequent HH survey. Locating the sampled HHs was more efficient with this information. Additionally, by importing the HH GPS coordinates, head of HH name, and HH descriptor that were collected at the time of the census into the HH survey data collection platform ensured that the HHs sampled were the HHs surveyed. This proved critical when multiple HHs were in the same structure.

The census activities had several important limitations. First, the duration was 6 days longer than anticipated due to a combination of factors including administrative challenges (e.g., vehicle rental commitment not being immediately fulfilled, personnel contract issues) and inclement weather (e.g., causing flooded roads/rivers to cross). Next, although near real-time data reporting was a strength of the census, operational challenges arose with respect to tablet use and data connectivity issues. Some issues were foreseen, such as working in remote locations where the tablets would need to be brought to a location with known cellular service to synchronize data to the server. Other issues were unforeseen, such as tablets with SIM cards and data plans that could not connect to the cell network. When this occurred, data from tablets that would not synchronize were transferred to different tablets using Wi-Fi Direct to synchronize, and subsequently data were submitted to the server. The tablet issues required staff in the field with technology skills to troubleshoot. Also, although the use of the georeferenced PDF maps on the tablets was a strength, it quickly drained the tablet batteries. External battery chargers were provided to each team, but more were required than anticipated. Teams were encouraged to use the provided paper maps and one tablet per team for navigation to preserve the battery of the other tablet to complete the census forms.

The census maps created at the grid cell EA level were helpful to field teams. However, gridded 1-km^2^ EAs did not match the terrain of landscape boundaries (rivers, mountains). Sometimes teams spent a long time identifying the best way to get to an area. This was anticipated, and the study area was also divided into 31 work zones that more accurately represented the physical geography. Unfortunately, only three maps could be loaded on the free version of PDF Maps^TM^ at a time. Because of the file size, teams preferred to load OpenStreetMap (OSM) maps than those with satellite imagery. Navigation errors and functionality could be improved by offline cached and tiled aerial and OSM imagery available in some apps now (e.g., ArcGIS Survey123, MapBox, SWMaps). Teams primarily identified HH populations during daytime hours (8 am–5 pm) and asked neighbors to verify the occupancy status of the unknown structures. It is possible that the estimated structures (3.67 persons per unknown HH) had a higher or lower number of people.

Finally, data from all teams were often not confirmed to be synchronized until late at night. With frequent electricity outages and data connectivity issues, this required overnight work to upload data and generate coverage maps to provide daily feedback to teams in the morning. This work was integral to the success of the census activities and directly influenced the quality of the data. Although a semiautomated ArcGIS Online workflow was created, it was not used as poor internet connectivity prevented field staff from accessing ArcGIS Online. In the future, robust planning for nighttime work and daily review should be considered.

Using maps with digitized structures for deployment, collecting ancillary data to aid in the return to sampled households, and providing daily feedback with updated coverage maps were novel methods that improved the quality of the population census and the sampling frame for the Artibonite HH survey. As advances are made in creating population estimates that incorporate multiple types of data (e.g., nighttime lights, cell phone usage, road networks, machine learning identification of structures), it is recommended to review all areas of low population before usage. In particular, because field teams may skip hard-to-reach areas thought to be uninhabited indicated by local guides (who may only know a limited area), overlays of digitized structures are critical field tools. If a census is not possible, when feasible, we recommend remotely digitizing structures and estimating occupancy. However, given that 9.7% of the censused structures were >30.4 m from digitized points, there also needs to be a strategy to include HHs not visible with satellite imagery in HH survey samples. Using a strategy such as a gridded method ensures that at least some of the HHs in the sparsest populated areas are counted. This is critical because people living in hard-to-reach areas may have disproportionately worse health outcomes than those in easier-to-reach areas that may be missed if more rigorous sampling frames are not used. When a census is conducted for a survey, it aids the community of practice to report on how using other sampling frames would have impacted the total number of HHs selected and survey planning.

## Supplemental Materials

10.4269/ajtmh.23-0891 Supplemental Materials
