# Supplementary material for: Evaluating Geospatial Sampling Frames with a Novel Field Census for a Malaria Household Survey in Artibonite, Haiti
Source: Am J Trop Med Hyg. 2024 Aug 13;111(4):770–9. doi: 10.4269/ajtmh.23-0891 (PMC11448545; doi:10.4269/ajtmh.23-0891)
Supplement: Supplemental Materials [file tpmd230891.SD1.pdf]

Supplementary Table 1. Summary of sampling frame methodologies.

#	Sampling Frame Method	Quality	Disadvantages
1.	Household Census:<br>• Map and list every HH in selected area.<br>• Select a simple random sample from HH list.	Gold Standard high quality data	Costly: in terms of time and personnel
2.	LandScan™ Population:<br>• Use population counts and recent DHS to estimate # of HHs<br>• Select grid based on estimated # of HHs	LandScan™ data is updated yearly and is available worldwide.	Does not address field level of how to select specific HHs. Produces 1 value per grid cell; survey team must still identify HHs and select.
3.	Digitization method:<br>• Use high resolution imagery to manually digitize all structures (add a point) >3 m^2<br>• Use past surveys to estimate proportion of structures that are HHs. Use DHS to calculate population	Unknown – medium<br><br>This is what is under evaluation for the future (to reduce cost and save time)	Requires imagery which has a cost to acquire. Requires advance time and training to digitize structures. If known, method assisted by % of structures that are HHs
4.	Census enumeration areas:<br>• Total population, number of structures, and number of HHs per small area<br>• Select EAs, then multiple ways to select HHs (census and simple random sample; skipping interval; segmentation)	Variable – reliability decreases with age of census. Error may be introduced by teams during HH selection.<br><br>This is what most people do now.	Census enumeration area limits often available at a cost or not digitally. Relies often on data 10+ years old.
